# Structural and Echocardiographic Abnormalities in Congenital Long QT Syndrome: A Review of the Literature

**DOI:** 10.3390/medicina62050829

**Published:** 2026-04-27

**Authors:** Austė Markevičiūtė, Patricija Lapinskaitė, Mariola Kovalevska, Audronė Vaitiekienė, Diana Rinkūnienė

**Affiliations:** 1 Faculty of Medicine, Medical Academy, Lithuanian University of Health Sciences, 44307 Kaunas, Lithuania; 2 Karoliniskiu Polyclinic, 04318 Vilnius, Lithuania; 3 Heart Centre, Lithuanian University of Health Sciences, 44307 Kaunas, Lithuania

**Keywords:** long QT syndrome, echocardiography, left atrial remodeling, speckle-tracking echocardiography, ventricular arrhythmias

## Abstract

Congenital Long QT Syndrome (LQTS) is a hereditary cardiac channelopathy defined by delayed ventricular repolarization and an elevated risk of life-threatening ventricular arrhythmias. Recent echocardiographic studies using speckle-tracking and strain imaging have identified subtle abnormalities in ventricular and atrial mechanics among LQTS patients, including reduced global longitudinal strain, impaired diastolic function, enlarged left atrial volumes and a consistently negative electromechanical window. These findings challenge the traditional concept of LQTS as solely an electrical disease and support evolving evidence of a subclinical cardiomyopathic phenotype. Left atrial remodeling, although less studied, may represent an underrecognized component of LQTS with potential implications for arrhythmia vulnerability and diastolic dysfunction. This review summarizes current evidence on electromechanical and structural cardiac involvement in congenital LQTS, highlights its diagnostic and clinical implications, and outlines future directions for research in this evolving field.

## 1. Introduction

Long QT syndrome (LQTS) is a genetically inherited cardiac channelopathy characterized by a prolonged ventricular repolarization, which results in a prolonged QT interval on electrocardiography (ECG) [1]. This conduction disorder is associated with an increased risk of ventricular arrhythmias (VAs) and sudden cardiac death (SCD), particularly among younger patients. The most extensive population-based study on the prevalence of congenital LQTS, published in 2009, estimated the prevalence of congenital LQTS to be approximately 1 in 2500 individuals [2]. However, this estimate was limited to infants with a prolonged heart rate (HR) corrected QT interval (QTc) and did not include asymptomatic carriers of pathogenic mutations. Consequently, the true prevalence of congenital LQTS may be much higher [3].

LQTS can be classified as either congenital (inherited) or acquired. Congenital LQTS results from mutations in genes encoding cardiac ion channels, whereas acquired LQTS may arise from electrolyte imbalances, comorbid medical conditions, or medications that alter the QT interval duration [4]. This review focuses solely on the congenital form of LQTS, given its distinct genetic etiology, clinical course, and potential for structural cardiac involvement.

More than 17 genetic mutations associated with congenital LQTS have been identified. The most prevalent forms are LQTS type 1 (LQT1), caused by mutations in the KCNQ1 gene; LQTS type 2 (LQT2), caused by mutations in the KCNH2 gene; and LQTS type 3 (LQT3), linked to mutations in the SCN5A gene [5]. These mutations alter ion channel function and prolong repolarization, potentially inducing structural changes at the cellular level that extend beyond electrophysiological disturbances.

Although LQTS was previously classified as a purely electrophysiological disorder, recent studies demonstrate that patients with LQTS may also exhibit subtle structural and mechanical cardiac alterations [6]. These observations extend beyond isolated ion channel dysfunction and include reports of increased left atrial (LA) volumes and a negative electromechanical window (EMW), particularly among symptomatic patients. While direct assessment of LA mechanics using strain imaging has not yet been systematically performed in LQTS cohorts, the potential for atrial involvement has been hypothesized and warrants further investigation [7].

While traditionally viewed strictly as an electrical disease, LQTS is increasingly recognized as a broader cardiomyopathic phenotype. Therefore, the overarching aim of this literature review is to comprehensively synthesize the current evidence regarding structural and mechanical cardiac alterations in patients with congenital LQTS. Specifically, the primary objectives of this study are to:

1. Detail the specific echocardiographic abnormalities identified in LQTS cohorts, including altered global longitudinal strain (GLS), EMW negativity, and LA remodeling;
2. Delineate genotype-specific structural and mechanical differences among the main LQTS subtypes;
3. Explore the proposed pathophysiological mechanisms linking electrical instability to structural myocardial remodeling;
4. Highlight the diagnostic, prognostic, and therapeutic implications of integrating advanced structural imaging into the clinical management of LQTS.

## 2. Methods

This article was designed as a narrative review summarizing current evidence on structural and echocardiographic abnormalities in congenital long QT syndrome. A comprehensive literature search was performed using major biomedical databases, including PubMed and Scopus, covering studies published up to February 2026.

Search terms included combinations of “long QT syndrome,” “LQTS,” “echocardiography,” “speckle-tracking echocardiography,” “strain,” “left atrial remodeling,” “electromechanical window,” and “ventricular arrhythmias.”

Additional relevant articles were identified through manual screening of reference lists of key publications and recent reviews., as well as major society guidelines. Priority was given to original research articles, cohort studies, and clinically relevant observational studies evaluating structural and functional cardiac changes in congenital LQTS.

## 3. Discussion

### 3.1. Clinical and Electrophysiological Features of Congenital LQTS

The clinical presentation of LQTS varies widely, from asymptomatic individuals to those experiencing syncope, seizures, or SCD. In symptomatic cases, manifestations are most often due to VAs, which present as palpitations. LQTS may also be initially suspected based on family history or incidental identification of a prolonged QT interval on ECG. Mutations in the KCNQ1, KCNH2, and SCN5A genes, corresponding to LQT1, LQT2, and LQT3, account for approximately 90% of genotype-positive inherited LQTS cases [8]. Each of these genotypes exhibits distinct clinical and electrophysiological characteristics. These features are summarized in Table 1.

LQTS type 1 results from a loss-of-function mutation in the KCNQ1 gene, which encodes the α-subunit—Kv7.1—of a cardiac potassium channel. In conjunction with the KCNE1 protein, four Kv7.1 subunits form a channel that conducts the slow delayed rectifier potassium current, Iks, during repolarization, facilitating potassium efflux and providing repolarization reserve. The Iks current is especially critical during adrenergic stimulation, such as exercise or emotional stress, as it enables repolarization to accelerate and the QT interval to shorten at elevated HR. Mutations in KCNQ1 disrupt the structure of the potassium channel, leading to a reduction or complete loss of Iks and prolonging repolarization. Consequently, the QT interval remains abnormally prolonged during tachycardia, increasing electrical instability and predisposing to VAs, including torsades de pointes. Patients with LQTS1 are therefore most susceptible to arrhythmic events, often manifesting as syncope or palpitations, during physical exercise or adrenergic stress. Adolescents and young adults are at particular risk, and in some cases, SCD may be the initial presentation [8,9].

LQTS type 2 is caused by a loss-of-function mutation in the KCNH2 gene, which encodes the α-subunit of the potassium channel protein, called Kv11.1. This channel mediates the “rapid delayed rectifier potassium current” or Ikr. Under normal circumstances, Ikr contributes to the rapid phase of repolarization, helping to terminate the action potential and restore the resting membrane potential. However, loss-of-function KCNH2 mutations reduce Ikr, slowing repolarization and prolonging the action potential duration, thereby prolonging the QT interval. Unlike Iks, Ikr functions largely independently of sympathetic stimulation [10]. Because of this, the QT interval in LQTS2 patients is less responsive to changes in HR, and repolarization remains vulnerable to abrupt sympathetic surges. As a result, arrhythmic events in LQT2 patients are often triggered by sudden auditory stimuli or emotional stress, which can provoke early afterdepolarizations and/or torsades de pointes [11]. Certain patient groups are particularly predisposed to VAs, including women, who exhibit a higher risk than men, especially during the postpartum period when hormonal changes and frequent sudden stimuli further destabilize repolarization [12].

**Figure 1:**
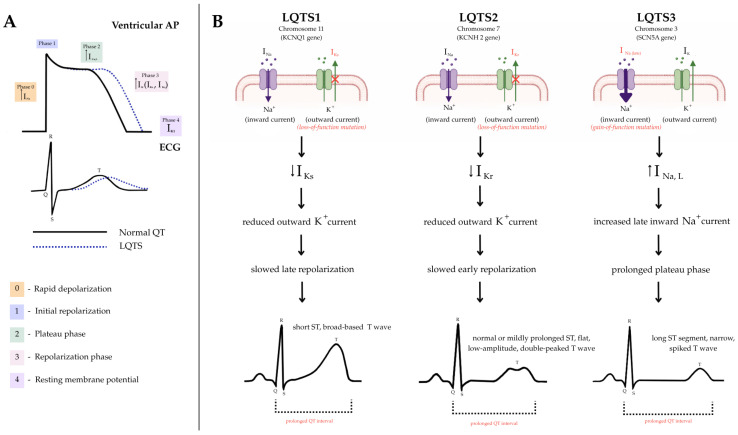
Pathophysiological mechanisms of congenital long QT syndrome: integration of ion channel dysfunction, action potential changes, and ECG manifestations. (**A**) Normal ventricular action potential and corresponding ECG, with illustration of delayed repolarization and QT interval prolongation in LQTS. (**B**) Genotype-specific mechanisms of LQTS: LQTS1 and LQTS2 are associated with reduced potassium currents, whereas LQTS3 is characterized by increased late sodium current, leading to action potential prolongation and characteristic ECG changes. ↑—increased channel current; ↓—decreased channel current. Abbreviations: AP—action potential; LQTS—long QT syndrome; I_Ks_—slow delayed rectifier potassium current; I_Kr_—rapid delayed rectifier potassium current; I_Na,L_—late sodium current; ECG—electrocardiogram.

LQTS type 3 is caused by a gain-of-function mutation in the SCN5A gene, which encodes the α-subunit of the cardiac sodium channel Nav1.5. Under normal physiological conditions, these channels briefly activate during the initial depolarization phase of the cardiac action potential and then rapidly inactivate, allowing repolarization to proceed normally. In LQT3, impaired channel inactivation leads to an enhanced late sodium current, called Ina, which prolongs the plateau phase and delays repolarization. In contrast to LQT1 and LQT2, where adrenergic stimulation is a key VA trigger, LQT3-related events typically occur at rest or during sleep, when increased parasympathetic activity and low HR further prolong repolarization [13]. A schematic overview of genotype-specific electrophysiological mechanisms linking ion channel dysfunction to action potential alterations and ECG manifestations is presented in Figure 1.

Although the clinical expression of congenital LQTS is highly variable, recognition of genotype-specific features, including typical arrhythmic triggers and associated risk profiles, is crucial for patient management. Diagnosis relies on a combination of clinical presentation, family history, characteristic T-wave morphology, and a prolonged QTc interval on the ECG, and is ultimately confirmed by genetic testing [14].

### 3.2. Structural and Echocardiographic Abnormalities in Congenital LQTS

Long considered a purely electrical disease, LQTS is now recognized to involve structural and mechanical cardiac changes. This recognition underscores the potential of advanced transthoracic echocardiographic (TTE) assessment to enhance patient risk stratification and improve understanding of disease pathophysiology. Standard TTE in LQTS patients typically shows normal chamber sizes and systolic function. However, detailed analyses using speckle-tracking echocardiography (STE) and strain imaging have identified subtle yet clinically relevant echocardiographic abnormalities [15].

A consistent observation in symptomatic LQTS patients is a negative electromechanical window (EMW), defined as the difference between the interval from QRS onset to aortic valve closure, measured by continuous-wave Doppler, and the QT interval from the ECG [16]. In a large study by Sugrue et al. involving 651 LQTS patients, nearly all exhibited negative EMW values, with symptomatic patients demonstrating significantly greater negativity (−52 ± 38 ms versus −18 ± 29 ms in asymptomatic patients) [17]. These findings are consistent with observations by Odening et al., supporting the concept of electromechanical reciprocity [6]. In their study, a more negative EMW was associated with a higher risk of VAs and SCD. However, several important limitations should be acknowledged. The study by Sugrue et al., although relatively large, represents a single-center cohort derived from a specialized referral population, which may introduce selection bias and limit generalizability to broader LQTS populations. In addition, a substantial proportion of patients were excluded due to suboptimal ECG or echocardiographic signal quality, particularly difficulties in accurately defining T-wave termination, highlighting potential challenges in EMW measurement in routine clinical practice. Moreover, EMW assessment requires precise alignment of Doppler-derived mechanical events with ECG intervals, which may introduce measurement variability and limit reproducibility in routine practice. Similarly, evidence from Odening et al. is largely based on integrative and relatively small or heterogeneous cohorts, and electromechanical parameters such as EMW are not yet standardized or routinely applied in clinical risk stratification. Taken together, although EMW is a promising marker of arrhythmic risk, methodological variability, lack of standardized thresholds, and limited external validation currently restrict its widespread clinical use. Recently published work by Deissler and colleagues highlighted several interesting future directions for EMW research. They proposed moving beyond a single global measurement toward segmental EMW assessment by integrating regional mechanical information from strain imaging with three-dimensional electrical mapping. Moreover, they emphasized the importance of evaluating temporal EMW variability, which may help detect high-risk patterns before arrhythmic events occur. Continuous monitoring using signals from Implantable cardioverter defibrillator (ICD) or wearable devices and assessing EMW dynamics as a marker of treatment response were also suggested as promising approaches to improve risk stratification in congenital LQTS [18]. Together, these findings suggest that EMW is one of the most promising echocardiographic-derived markers in LQTS, with consistent associations with arrhythmic risk. However, its use remains largely limited to specialized centers, and further standardization is required before routine clinical implementation.

One of the earliest systematic evaluations, conducted by the Mayo Clinic LQTS cohort in 2013, analyzed 216 patients. Notably, 25% exhibited at least one abnormal echocardiographic finding despite normal systolic function on standard TTE imaging. The most common abnormality was an increased LA volume index, followed by enlargement of the left or right ventricle and grade I-II diastolic dysfunction. The LA volume index was statistically higher in patients with a history of arrhythmic events (24.4 ± 5.5 mL/m^2^ versus 22.3 ± 6.1 mL/m^2^, *p* = 0.02) [19]. However, this study was retrospective and included only patients who underwent echocardiography at a tertiary referral center, which may introduce selection bias and limit generalizability to the broader LQTS population. In addition, the majority of findings were subtle and within near-normal ranges, and the clinical significance of these subclinical abnormalities remains uncertain. Similar findings were reported by Leren et al. in 2015, who investigated 192 genotyped LQTS patients and demonstrated modest differences in left ventricular (LV) GLS between LQTS patients and healthy controls (−22.1 ± 2.1% versus −23.0 ± 2.0%, *p* = 0.01), as well as impaired diastolic function, reflected by lower e′ velocity (10.7 ± 2.7 cm/s versus 12.5 ± 2.0 cm/s, *p* < 0.001), and enlarged LA volumes (30 ± 8 mL/m^2^ versus 26 ± 5 mL/m^2^, *p* = 0.01) [20]. However, it should be noted that these differences, although statistically significant, are relatively small in absolute terms and largely remain within normal physiological ranges, which may limit their direct clinical relevance. Additionally, most strain studies are cross-sectional, with limited longitudinal data, making it unclear whether these subtle alterations progress over time. Moreover, advanced strain imaging is highly dependent on image quality and technical expertise, and was not feasible in all patients, highlighting limitations in reproducibility, standardization, and broader applicability—particularly in non-specialized or lower-volume centers where such techniques may not be routinely available. Subsequent studies employing advanced strain imaging have also further clarified regional and transmural abnormalities. Haugaa et al. demonstrated prolonged myocardial contraction duration in LQTS mutation carriers (445 ± 45 ms versus 390 ± 40 ms in controls, *p* < 0.001), with symptomatic patients exhibiting even longer durations [21]. Charisopoulou et al. extended these findings to exercise conditions, documenting a reversed apico-basal myocardial relaxation sequence in LQTS carriers with prior events, especially in LQT1 and LQT2 subtypes [15]. However, this study was conducted in a relatively small cohort (n = 47) and included only patients in whom complete segmental strain analysis was feasible, introducing potential selection bias related to image quality and limiting external validity, while the assessment of temporal strain parameters (e.g., tESR and apico-basal relaxation gradients) further depends on vendor-specific software and frame rates, which remain insufficiently standardized and complicate comparisons across studies. Taken together, these observations suggest alterations in myocardial mechanics rather than overt structural abnormalities, although their clinical applicability remains limited by the heterogeneity of available data and lack of methodological standardization. Most studies to date have focused on ventricular mechanical abnormalities in LQTS; therefore, the potential involvement of the atria remains largely unexplored. Nevertheless, several observations suggest that atrial remodeling may also be present and clinically relevant. As described above, increased LA volumes have been reported in both the Mayo Clinic cohort and the study by Leren et al., indicating that atrial structural changes can and do occur in patients with congenital LQTS [19,20]. However, beyond these findings, systematic investigation of atrial mechanics, particularly using deformation imaging, has not yet been performed in this population. Comprehensive assessment of LA strain, including reservoir, conduit, and contractile functions, could provide deeper insights into atrial electromechanical remodeling, help detect early diastolic dysfunction, and refine risk stratification, especially in patients with borderline QT prolongation.

Recent studies have also further refined the understanding of genotype-specific mechanical remodeling in LQTS. Bileišienė et al. demonstrated that LQT2 was associated with significantly longer mid-cavity radial contraction duration, with a trend toward increased mechanical dispersion, compared to LQT1 [22]. Similarly, Marwick and colleagues reported that LA enlargement occurred in approximately 20% of LQTS patients and suggested that post-systolic shortening may contribute to diastolic dysfunction [7]. Patients with LQT1 and LQT2 more frequently exhibit apical contractile prolongation and reversed relaxation sequences during stress [15], whereas LQT3 (SCN5A mutation) has been associated with features overlapping dilated cardiomyopathy phenotypes in certain families [23,24]. These genotype-specific differences further support the presence of a distinct mechanical phenotype in LQTS, characterized by regional and temporal abnormalities in myocardial contraction [25].

Importantly, evidence from a comprehensive meta-analysis indicates that LQTS patients exhibit consistent electromechanical abnormalities compared to controls, including prolonged contraction duration, increased mechanical dispersion, more negative EMW, and impaired diastolic function despite preserved LVEF. Notably, these abnormalities are more pronounced in symptomatic patients, in whom contraction duration, mechanical dispersion, and EMW show stronger associations with cardiac events than QTc. In this context, contraction duration has emerged as one of the most robust predictors, with reported thresholds (e.g., ≥430 ms) demonstrating better discriminatory performance for cardiac events than both EMW and QTc in pooled analyses. These findings suggest that selected echocardiographic parameters—particularly contraction duration, EMW, and mechanical dispersion—may complement QTc in arrhythmic risk assessment, especially in higher-risk or symptomatic patients [26]. To facilitate interpretation and provide a structured overview of these findings, key echocardiographic parameters in congenital LQTS, including their measurement, main abnormalities, and potential clinical implications, are summarized in Table 2.

Considering these findings, a more structured and clinically oriented echocardiographic approach may be proposed. In all patients with suspected or confirmed congenital LQTS, standard transthoracic echocardiography should be performed to exclude structural heart disease and to assess chamber dimensions, left ventricular systolic function, and left atrial size, with particular attention to diastolic indices such as transmitral inflow and tissue Doppler e′ velocity. Where expertise and software availability permit, advanced techniques, including STE, may be applied to evaluate GLS, regional contraction patterns, and mechanical dispersion. In selected cases, additional electromechanical parameters—such as contraction duration (measured from QRS onset to the end of post-ejection velocity), mechanical dispersion (defined as the standard deviation of contraction duration), aortic valve closure timing (from QRS onset to peak systolic strain) and the electromechanical window (calculated as the difference between the end of the QT interval and aortic valve closure)—may provide further insight into myocardial electromechanical coupling. The use of these advanced parameters may be particularly justified in patients with borderline QT prolongation, unexplained syncope, discordance between genotype and clinical phenotype, or suspected increased arrhythmic risk despite inconclusive standard evaluation. They may also be considered in specific populations, such as physically active individuals or athletes, as well as in specialized or research settings where longitudinal assessment is feasible. However, despite growing evidence, these techniques remain insufficiently standardized and are not yet incorporated into routine risk stratification algorithms. Therefore, echocardiography should currently be regarded as a complementary tool for phenotypic characterization and risk refinement rather than a primary determinant of clinical decision-making.

### 3.3. Pathophysiological Mechanisms

The pathophysiological relationship between LQTS and LA remodeling remains poorly understood, although several hypotheses have been proposed to explain it. The IKs potassium channel, encoded by the KCNQ1 gene, is essential for both ventricular and atrial repolarization [8]. Dysfunction of this channel may directly alter atrial myocyte electrophysiology and calcium handling, promoting fibrotic remodeling and structural changes over time. In addition, the chronic electrical instability inherent to LQTS—even when manifesting only as frequent premature depolarizations—could cause sustained mechanical stress and increased hemodynamic load on the atrial myocardium, further driving remodeling processes [22]. Finally, a broader subclinical myocardial phenotype has been proposed, as supported by findings from Odening et al., who reported increased ventricular trabeculation on cardiac magnetic resonance (CMR) in patients with congenital LQTS [6]. However, this finding should be interpreted with caution, as trabeculation patterns are highly variable and not uniformly defined. These observations may suggest the presence of subtle myocardial alterations beyond electrical abnormalities. In this context, the LA, due to its structural and hemodynamic vulnerability, may also be susceptible to such potential subclinical changes, and its remodeling could represent an underrecognized component of the LQTS phenotype.

In addition to the direct effects of IKs or IKr channel dysfunction on atrial myocytes, chronic electrical instability induces sustained mechanical stress and hemodynamic overload in the thin-walled LA, which promotes calcium-handling abnormalities, fibrosis, and remodeling. Odening et al. proposed that electromechanical reciprocity, together with subclinical myocardial features such as ventricular trabeculation observed on CMR, may represent a fundamental component of the LQTS phenotype across all genotypes [6]. Additionally, in LQT3, gain-of-function mutations in SCN5A may contribute to structural myocardial changes by enhancing late sodium current and inducing secondary fibrotic processes [23,24]. These mechanisms may account for the occurrence of LA enlargement and diastolic dysfunction even in cases with only mildly prolonged QTc, potentially preceding overt arrhythmic events.

### 3.4. Diagnostic Implications

The diagnosis of congenital LQTS relies on an integrated assessment of clinical, ECG, genetic, and structural parameters.

Patients with congenital LQTS may present with syncope, seizures, or cardiac arrest due to torsades de pointes, although many remain asymptomatic. Clinical events are often triggered by genotype-specific factors, and a family history of SCD at an early age supports the diagnosis of LQTS. Syncope is a key risk marker for SCD, particularly within the first year after the event, while additional risk factors, including electrolyte disbalance, medications, comorbidities, and genetic modifiers, may further increase the risk of VAs [27]. Molecular autopsy studies have demonstrated that pathogenic variants associated with LQTS may be identified in a proportion of sudden infant death syndrome and sudden unexplained death cases, particularly in young individuals. These findings highlight the importance of early detection and genetic evaluation in individuals at risk [28].

According to the 2022 ESC Guidelines, LQTS should be diagnosed when QTc ≥ 480 ms on repeated 12-lead ECGs is found, with or without symptoms, or when the LQTS diagnostic score exceeds 3. The presence of a pathogenic mutation confirms the diagnosis regardless of QT duration, whereas a QTc ≥ 460 ms but < 480 ms, combined with unexplained arrhythmic syncope, should prompt consideration for diagnostic evaluation [29]. In both cases a secondary cause for QT prolongation should be excluded. The LQTS diagnostic score (Schwartz score), which can be seen in Table 3, integrates ECG findings, clinical history, and genetic data, and remains a useful tool in borderline cases, particularly when QT prolongation is modest or intermittent [30].

Within this integrated diagnostic approach, ECG remains the cornerstone for the detection of QT prolongation and characteristic T-wave morphology. Importantly, a single ECG recording may be insufficient, and repeated (serial) ECGs are often required to confirm persistent QT prolongation, particularly in patients with borderline or variable. Accurate QT interval measurement is essential and should preferably be performed manually, typically in leads II, V2 or V3 using the tangent method to avoid inclusion of U-waves. The longest measurable QT interval across leads should be considered, as inter-lead variability may be significant. The QT interval should be averaged over at least three consecutive beats during stable sinus rhythm. Since the QT interval changes with varying HR, correction formulas are used to derive QTc. Bazett’s formula is most commonly applied in clinical practice. However, it may overestimate QTc at higher HR and underestimate it in bradycardia. In such cases alternative formulas, such as Fridericia or Framingham, may provide more accurate estimates. Although QTc is central to diagnosis, it should not be interpreted in isolation, as some patients with genetically confirmed LQTS may present with normal QTc values, whereas mild prolongation may also be observed in healthy individuals. 24-h Holter monitoring can be considered in selected patients to evaluate fluctuations in QT duration over time. However, accurate assessment is possible only during stable sinus rhythm and may be unreliable in the presence of arrhythmias or artifacts. Any suspected QT prolongation detected on Holter should be verified on a standard 12-lead ECG. Beyond QT interval prolongation, several additional ECG features may support the diagnosis and indicate increased arrhythmic risk. These include T-wave abnormalities such as notching or alternans, as well as intermittent sinus pauses, particularly in LQT3. In rare cases, ventricular arrhythmias may occur, most notably torsades de pointes [31]. In addition, dynamic or provocative testing may provide further diagnostic value, particularly in patients with borderline or normal QTc intervals at rest. Exercise testing, especially QTc assessment during the 4-min recovery phase, is the most widely used method and can unmask abnormal repolarization, particularly in LQT1 patients [32]. Similarly, simple bedside maneuvers, such as the abrupt standing test, can reveal impaired QT adaptation to sudden HR acceleration. While the QT interval normally shortens with increasing HR, patients with LQTS often demonstrate a blunted or paradoxical response, accompanied by characteristic T-wave abnormalities. In some cases, QTc prolongation may persist even after the HR normalizes, a phenomenon known as “QT stunning” [33]. Pharmacological provocative testing with epinephrine had also been used in the past to provoke paradoxical QT prolongation, particularly in LQT1 patient groups. However, its clinical utility is limited by variable reproducibility and the risk of false-positive results, partly related to the fusion of T and U waves and challenging test interpretation. For this reason, current ESC guidelines no longer recommend routine use of epinephrine testing in the diagnostic evaluation of LQTS [29].

Beyond electrical assessment, structural and mechanical evaluation is increasingly recognized as another valuable tool in LQTS assessment. Echocardiography with LA strain analysis provides functional insights beyond simple volumetric measurements and enables early detection of LA dysfunction that may otherwise remain subclinical. CMR can exclude alternative causes of LA structural changes, such as myocarditis or extensive fibrosis, thereby strengthening the link between observed remodeling and the primary channelopathy [34,35].

### 3.5. Therapeutic and Prognostic Considerations

Management of congenital LQTS relies on a combination of general preventive strategies, genotype-specific therapy, and individualized risk stratification. All patients should avoid QT-prolonging medications, which can be seen in Table 4, correct electrolyte imbalances, and minimize genotype-specific triggers, which were discussed above. In addition, lifestyle modifications represent an essential component of management and should be tailored according to genotype-specific risk profiles. Patients with LQT1 are advised to avoid strenuous exercise, particularly swimming, whereas those with LQT2 should minimize exposure to sudden auditory stimuli and emotional stress. In LQT3, events typically occur at rest or during sleep, and careful HR monitoring may be relevant.

Beta-blockers (preferably non-selective, such as nadolol or propranolol) are the first-line treatment in all patients with documented QT prolongation [36]. However, increasing evidence suggests that beta-blocker efficacy is not uniform across this drug class and may differ according to both pharmacokinetic properties and LQTS genotype. In particular, nadolol, a long-acting non-selective beta-blocker, provides more consistent protection, likely due to stable plasma concentrations and sustained β_1_/β_2_ blockade. In contrast, shorter-acting agents such as propranolol may result in less stable protection due to fluctuations in drug levels. Genotype-specific differences are also relevant. In LQT1, where arrhythmias are primarily adrenergically mediated, nadolol demonstrates the most pronounced benefit. In LQT2 and LQT3, beta-blocker efficacy appears reduced, likely due to the greater role of early afterdepolarizations, although nadolol remains the preferred agent. Notably, selective β_1_-blockers may be less effective in LQT3, where bradycardia-dependent arrhythmias predominate [37]. In addition to beta-blocker therapy, genotype-specific pharmacological approaches may be considered in selected patients. Mexiletine and flecainide are explicitly indicated for LQT3 to reduce the late sodium current and shorten QT duration. In contrast, sodium channel blockers such as flecainide or ranolazine are not recommended in both LQT1 and LQT2. Additional genotype-directed therapies have been explored, including potassium supplementation in selected patients with LQT2 to enhance repolarization reserve, although clinical evidence remains limited and individualized decision-making is required.

ICD implantation is recommended for patients with aborted cardiac arrest or recurrent VA-induced syncopes despite optimal pharmacological therapy, and it may be considered in high-risk asymptomatic patients based on genotype and QTc duration. ICD therapy is particularly relevant in high-risk subgroups, such as patients with LQT3, who may exhibit a reduced response despite treatment with maximally tolerated doses of beta-blockers and are more likely to experience arrhythmic events at rest or during sleep. In these patients, device therapy may play a central role in preventing SCD. However, the decision to implant an ICD requires careful individualized assessment, as device therapy is associated with potential complications, including inappropriate shocks, lead-related issues, and a significant psychosocial burden, particularly in younger patients [38]. In addition, recently validated prediction models may further refine prognostic assessment in congenital LQTS. Mazzanti et al. validated the 1-2-3-LQTS-Risk model, which estimates the baseline 5-year risk of life-threatening arrhythmic events according to genotype and QTc duration. This model may help identify patients who are most likely to benefit from ICD implantation while reducing unnecessary device therapy [39].

Left cardiac sympathetic denervation (LCSD) is recommended as an adjunctive therapy in patients with recurrent VAs despite optimal pharmacological treatment or ICD therapy, or when these therapies are contraindicated or not tolerated. LCSD reduces arrhythmic syncope and decreases the overall frequency of VAs in congenital LQTS [40]. In addition to its adjunctive role, LCSD has emerged as an important strategy for arrhythmic risk reduction, particularly in younger patients, where it may be considered prior to ICD implantation. Its antiarrhythmic effect is mediated through attenuation of sympathetic input to the heart and an increase in the ventricular fibrillation threshold, which has been associated with a substantial reduction in cardiac events. This effect appears particularly relevant in patients with high adrenergic susceptibility, such as those with LQT1 and LQT2, as well as in individuals with recurring arrhythmic events despite beta-blocker therapy. Although LCSD is not curative, it may, in selected cases, serve as an alternative to device therapy, particularly in patients who are intolerant to beta-blockers or at risk of ICD-related complications. Carefully selected low-risk patients, typically characterized by moderate QTc prolongation and low arrhythmic burden, may benefit from this approach, although such decisions require individualized risk assessment.

Emerging therapeutic strategies, including gene therapy and targeted molecular approaches, are also currently being actively investigated in congenital LQTS. Recent preclinical studies have demonstrated the potential of gene-based approaches to directly correct the underlying ion channel dysfunction. In LQT1, suppression-replacement strategies targeting the KCNQ1 gene have shown the ability to normalize repolarization by restoring functional channel expression, effectively reversing the prolonged action potential phenotype in patient-derived cardiomyocytes [41]. Similarly, advances in genome editing technologies, including CRISPR-based and prime editing approaches, have enabled precise correction of pathogenic variants in genes such as KCNH2 and SCN5A, which are implicated in LQT2 and LQT3, respectively. These strategies aim not only to mitigate electrophysiological abnormalities but to provide mutation-specific, potentially curative interventions at the DNA level [42]. Preclinical studies suggest that correction of underlying ion channel dysfunction at the genetic level may reduce arrhythmic risk and offer future genotype-specific treatment options. In addition, patient-specific cellular models may enable more personalized therapeutic strategies. Despite these promising developments, current evidence remains limited to experimental and early translational studies, and significant challenges, including delivery methods, long-term safety, and off-target effects, must be addressed before clinical implementation [43].

### 3.6. Future Directions and Research Needs

Future studies should focus on clarifying the relationship between congenital LQTS and structural cardiac remodeling, particularly the extent of atrial involvement. The severity of LA structural changes may serve as a novel risk marker in congenital LQTS. Further research should use advanced multimodality imaging techniques, such as STE, to detect early and subtle myocardial changes across different LQTS genotypes. Long-term follow-up studies are also needed to determine whether these structural and functional abnormalities precede arrhythmic events or develop as secondary adaptations to chronic electrical instability. Ultimately, integrating imaging, electrophysiological, and genetic data into comprehensive risk models may improve risk prediction and enable a more personalized management of LQTS.

Prospective multicenter studies employing STE to systematically evaluate LA reservoir, conduit, and contractile strain functions are required. Integration of artificial intelligence for automated segmental and temporal EMW variability analysis, combined with wearable devices and ICD telemetry, could revolutionize real-time risk prediction and treatment monitoring [18,44]. In addition, recent studies suggest that automated, quantitative analysis of T-wave morphology may further refine risk stratification by identifying genotype-specific electrocardiographic “fingerprints” associated with cardiac events, thereby overcoming the limitations of subjective visual ECG interpretation [45].

## 4. Conclusions

1. Congenital long QT syndrome (LQTS) extends beyond an electrophysiological disorder—emerging data indicate the presence of subtle yet significant structural and functional cardiac remodeling, involving both ventricles and atria.
2. The principal findings of the reviewed literature suggest that negative electromechanical window, prolonged contraction duration, increased mechanical dispersion, mildly reduced global longitudinal strain, impaired diastolic indices, and enlarged left atrial volumes may represent measurable manifestations of this broader electromechanical phenotype.
3. A novel contribution of this review is the focused integration of currently available evidence on echocardiographic and structural abnormalities in congenital LQTS, with particular emphasis on genotype-related mechanical differences and the potential role of left atrial remodeling as an underrecognized component of the disease phenotype.
4. Among currently studied imaging markers, contraction duration, mechanical dispersion, and electromechanical window appear the most promising for refining arrhythmic risk assessment beyond conventional ECG-based evaluation, although their clinical use remains limited by insufficient standardization and external validation.
5. Future multicenter, genotype-specific, and longitudinal studies are required to determine whether integration of echocardiographic, electrocardiographic, and genetic data can improve individualized diagnosis, risk stratification, and management in congenital LQTS.

## Figures and Tables

**Table 1:** Key Subtypes of Congenital Long QT Syndrome and Their Clinical Characteristics.

LQTS Type	Gene	Mutated Protein (Channel)	Current Affected	Mutation Type	Clinical Triggers/Features
LQT1	KCNQ1	α-subunit of potassium channel (Kv7.1)	↓Iks (slow delayed rectifier potassium current)	Loss-of-function	Physical exercise, adrenergic stress. Adolescents and young adults are at particular risk.
LQT2	KCNH2	α-subunit of potassium channel (Kv11.1)	↓Ikr (rapid delayed rectifier potassium current)	Loss-of-function	Sudden auditory stimuli, emotional stress. Higher risk in women, especially during the postpartum period.
LQT3	SCN5A	α-subunit of sodium channel (Nav1.5)	↑Ina (enhanced late sodium current)	Gain-of-function	Rest or sleep (associated with increased parasympathetic activity and low heart rates).

LQTS—long QT syndrome; Iks—slow delayed rectifier potassium current; Ikr—rapid delayed rectifier potassium current; Ina—sodium current. ↑—increased channel current; ↓—decreased channel current.

**Table 2:** Echocardiographic parameters in long QT syndrome: measurement, main findings, and potential clinical relevance.

Parameter	Measurement/Definition	Main Findings in LQTS	Potential Clinical/Prognostic Relevance
Global longitudinal strain (GLS)	Speckle-tracking derived measure of longitudinal LV deformation	Mildly reduced vs. healthy controls despite preserved LVEF	May reflect subclinical myocardial dysfunction; limited standalone prognostic value
Mechanical dispersion (MD)	Standard deviation of time to peak longitudinal systolic strain across all left ventricular segments (typically 16-segment model)	Increased in LQTS; higher in symptomatic than in asymptomatic patients	Associated with electrical heterogeneity and arrhythmic risk
Contraction duration (CD)	Time from onset of R wave to the end of post-ejection velocity	Prolonged in LQTS, especially symptomatic patients	Strong predictor of cardiac events; may outperform QTc in some studies (e.g., ≥430 ms)
Electromechanical window (EMW)	Time difference between the end of QT interval and the onset of aortic valve closure	More negative in LQTS; markedly negative in symptomatic patients	Independent predictor of arrhythmic events; potential incremental value beyond QTc
Aortic valve closure timing (QAoC)	Difference between QRS onset to peak systolic strain or valve closure	Prolonged in LQTS	Reflects delayed mechanical systole; contributes to EMW
Left atrial (LA) volume index	LA volume indexed to body surface area	Mildly increased in some LQTS cohorts	May indicate early structural remodeling; unclear prognostic value
LA strain (reservoir, conduit, contractile	Speckle-tracking assessment of atrial deformation	Not systematically studied in LQTS	Potential marker of early atrial remodeling; clinical significance unknown
Diastolic indices (E/A, e′, IVRT)	Doppler-based assessment of LV filling and relaxation	Subtle diastolic impairment (↓E/A, ↓e′, ↑IVRT)	Suggest early diastolic dysfunction; prognostic role not well established

GLS—global longitudinal strain; MD—mechanical dispersion; CD—contraction duration; EMW—electromechanical window; QAoC—aortic valve closure timing; LA—left atrium; LV—left ventricle; LVEF—left ventricular ejection fraction; QTc—corrected QT interval; E/A—early to late diastolic transmitral flow velocity ratio; e′—early diastolic mitral annular velocity; IVRT—isovolumetric relaxation time; LQTS—long QT syndrome. ↑—increased channel current; ↓—decreased channel current.

**Table 3:** Clinical and electrocardiographic criteria used in the Schwartz score for congenital LQTS diagnosis.

		Points
	**Electrocardiographic** **findings**	
A	QTc	
	≥480 ms	3
	460–479 ms	2
	450–459 ms (in males)	1
B	QTc 4th minute of recovery from exercise stress test ≥480 ms	1
C	Torsades de pointes	2
D	T wave alternants	1
E	Notched T wave in 3 leads	1
F	Low heart rate for age	0.5
	**Clinical history**	
A	Syncope:	
	With stress	2
	Without stress	1
B	Congenital deafness	0.5
	**Family history**	
A	Family members with definite LQTS	1
B	Unexplained SCD age 30 among immediate family members	0.5

QTc—corrected QT interval; LQTS—long QT syndrome; SCD—sudden cardiac death.

**Table 4:** Common classes of medications associated with QT interval prolongation and their examples.

Drug Class	Examples
Antiarrhythmics	Amiodarone, sotalol, dofetilide, procainamide, quinidine, flecainide
Antipsychotics	Haloperidol, ziprasidone, quetiapine, thioridazine, olanzapine, risperidone, droperidol
Antidepressants	Amitriptyline, imipramine, citalopram, trazodone, venlafaxine
Antibiotics	Macrolides, fluoroquinolones
Opioids	Methadone, buprenorphine, oxycodone, tramadol
Antiemetics	Metoclopramide, 5-HT_3_ antagonists
Anticonvulsants	Lamotrigine, topiramate, gabapentin
Muscle relaxants	Tizanidine, eperisone
NSAIDs	Ketorolac, celecoxib, diclofenac
Other agents	Sumatriptan, cisapride

NSAIDs—non-steroidal anti-inflammatory drugs; 5-HT_3_—5-hydroxytryptamine type 3 receptor; QT—QT interval.

## Data Availability

No new data were created or analyzed in this study.

## Abbreviations

The following abbreviations are used in this manuscript:

LQTS	Long QT syndrome
ECG	Electrocardiography
VA	Ventricular arrhythmias
SCD	Sudden cardiac death
HR	Heart rate
QTc	Corrected QT interval
LQT1	LQTS type 1
LQT2	LQTS type 2
LD LQT3	LQTS type 3
LA	Left atrium
EMW	Electromechanical window
TTE	Transthoracic echocardiographic
STE	Speckle-tracking echocardiography
ICD	Implantable cardioverter defibrillator
LV	Left ventricle
GLS	Global longitudinal strain
CMR	Cardiac magnetic resonance
LCSD	Left cardiac sympathetic denervation
